# CircPVT1: a pivotal circular node intersecting Long Non-Coding-PVT1 and c-MYC oncogenic signals

**DOI:** 10.1186/s12943-022-01514-y

**Published:** 2022-01-28

**Authors:** Alina Catalina Palcau, Valeria Canu, Sara Donzelli, Sabrina Strano, Claudio Pulito, Giovanni Blandino

**Affiliations:** 1 Oncogenomic and Epigenetic Unit, IRCCS, Regina Elena National Cancer Institute, Via Elio Chianesi, 53, 00144 Rome, Italy; 2 SAFU Laboratory, Department of Research, Advanced Diagnostic, and Technological Innovation, IRCCS Regina Elena National Cancer Institute, Rome, Italy

**Keywords:** Non-coding RNA, circular RNA, circPVT1, PVT1, c-MYC, sponge, peptide

## Abstract

The role of circular RNAs in oncogenesis has begun to be widely studied in recent years, due to the significant impact that these molecules have in disease pathogenesis, as well as their potential for the future of innovative therapies. Moreover, due to their characteristically circular shape, circular RNAs are very resistant molecules to RNA degradation whose levels are easily assessed in body fluids. Accordingly, they represent an opportunity for the discovery of new diagnostic and prognostic markers in a wide range of diseases. Among circular RNAs, circPVT1 is a rather peculiar one that originates from the circularization of the exon 2 of the PVT1 gene that encodes a pro-tumorigenic long non-coding RNA named lncPVT1. There are a few examples of circular RNAs that derive from a locus producing another non-coding RNA. Despite their apparent transcriptional independence, which occurs using two different promoters, a possible synergistic effect in tumorigenesis cannot be excluded considering that both have been reported to correlate with the oncogenic phenotype. This complex mechanism of regulation appears to also be controlled by c-MYC. Indeed, the PVT1 locus is located only 53 Kb downstream c-MYC gene, a well-known oncogene that regulates the expression levels of about 15% of all genes. Here, we review circPVT1 origin and biogenesis highlighting the most important mechanisms through which it plays a fundamental role in oncogenesis, such as the well-known sponge activity on microRNAs, as well as its paradigmatic interactome link with lncPVT1 and c-MYC expression.

## Background

The ENCODE project has clearly reported that most human genomes, exactly 98%, encodes RNAs with apparently no coding potential [1]. This class of RNAs is called non-coding RNAs (ncRNAs). Their biological role was underestimated for several years, however only in the last decades their pivotal regulatory activity on gene expression has been demonstrated. Their aberrant expression has been shown to promote disease progression and cancer. Indeed, ncRNAs participate in controlling the expression of key tumour suppressor and tumour-promoting genes thus being often associated with tumour development and progression. For this reason, ncRNAs studies have tremendously increased in order to best elucidate the molecular mechanisms by which they act within the cell.

There are two categories of non-coding RNAs, the housekeeper non-coding RNAs and the regulatory non-coding RNAs. The first one includes the well-known ribosomal RNAs (rRNAs), transfer RNAs (tRNAs) or small-nuclear RNAs (snRNAs) that are expressed constitutively. Long non-coding RNAs (lncRNAs) that are longer than 200 nucleotides (nt) and those ncRNAs with less than 200 nucleotides (nt) such as microRNAs (miRNAs), piwi-associated RNAs (piRNAs) and small-interfering RNAs (siRNAs) can be included in the regulatory ncRNAs category.

Regulatory non-coding RNAs are extensively involved in the regulation of several pathways of cell physiology. Indeed, they have been found to regulate gene expression, mRNAs stability, interact with proteins, mediate gene silencing or intervene at the epigenetic level [2]. Aberrant expression of some regulatory non-coding RNAs has been associated with the development of several diseases [3], such as cancer [4]. Considering the key role that this class of non-coding RNAs have in cells, they represent a promising feature that needs to be thoroughly studied in order to understand the mechanisms leading to cancer development and find alternative treatments that have so far been unexplored.

Interestingly, in recent years circular RNA (circRNA), a new class of ncRNAs has emerged. For several years the world of circRNA has remained unexplored. Traditional methods for DNA/RNA detection such as electrophoretic or size mobility shift assays did not allow detecting circRNAs molecules. The peculiar circular structure allows them to be resistant to the exonuclease activity of RNase R since they do not present any 3’ or 5’ polarity [5]. They were originally identified in plant viroids and hepatitis delta virus and were considered splicing errors [6, 7]. Later, it has been demonstrated that they are actually actively involved in biological processes [8, 9]. Circular RNAs are molecules covalently closed and do not present any 3’ or 5’ polarity. For this reason, they were not considered in the transcriptomic polyadenylated RNA profiling [10]. The closed structure makes them very resistant to RNA degradation than their linear counterpart, and in some cases, these RNA molecules are also more abundant than the linear counterpart.

**Fig. 1:**
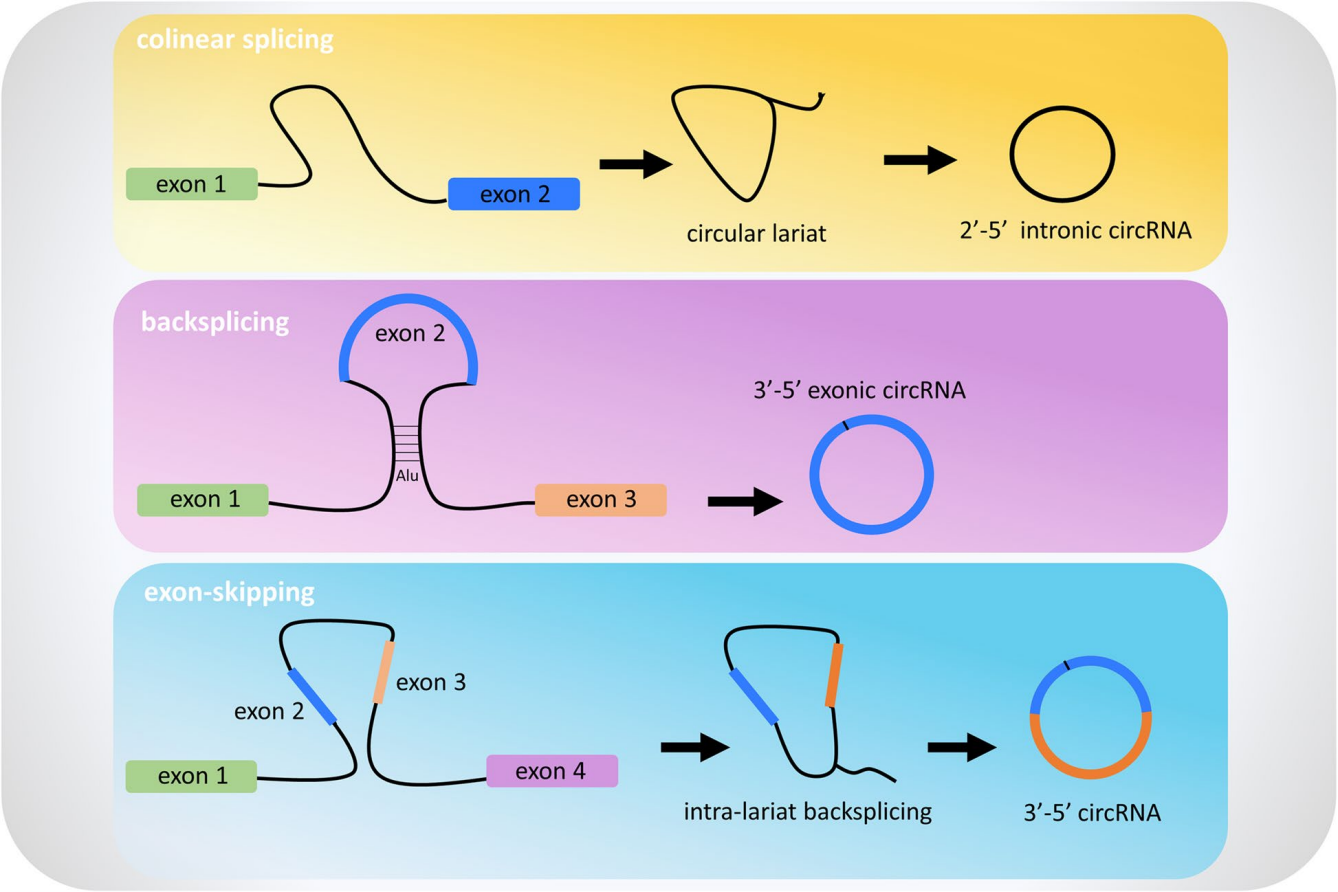
CircRNAs biogenesis. **a** After colinear splicing, the intron excised from a multi exon gene results in a 2’-5’ linked lariat that cannot be degraded but can be processed to become a stable 2’-5’circRNA. **b** Backsplicing process occurs when in a nascent mRNA, the 5’ end of an exon joins with its upstream 3’ end leading to the formation of a 3’-5’ exonic cirRNA. **c** During alternative splicing of linear mRNA, the excised exon is substrate for intralariat backsplicing leading to circRNA formation

Circular RNAs can be generated following colinear splicing, from a process called back-splicing or from exon skipping involving the spliceosome machinery. In 1999, Holdt L. M. and colleagues proposed three models to describe circular RNA biogenesis [11] (Fig. 1). The most frequent circRNA biogenesis model is backsplicing, but whether it happens more co-transcriptionally or post-transcriptionally is yet to be established. In both cases, in 70% of cases, circularization process requires spliceosome machinery [11].

In this review, we provide insight on circular RNAs characteristics by depicting their functions. We also describe the emerging role of the circPVT1/lncPVT1/c-MYC paradigmatic network in cancer disease detailing the molecular mechanisms underlying its pro-tumorigenic activities.

## Circular-RNAs mechanism of action

In early 1990 Vogelstein’s group identified abnormally spliced transcripts including circular RNAs for the tumor suppressor gene, DCC in normal and cancer cells in rodents and humans [12]. Since the discovery of the existence of circular RNAs, several studies have been carried out to investigate their mechanisms of action, revealing their involvement in many biological processes [13–15]. Alterations at the circular RNAs expression level can indeed be associated with tumorigenesis.

**Fig. 2:**
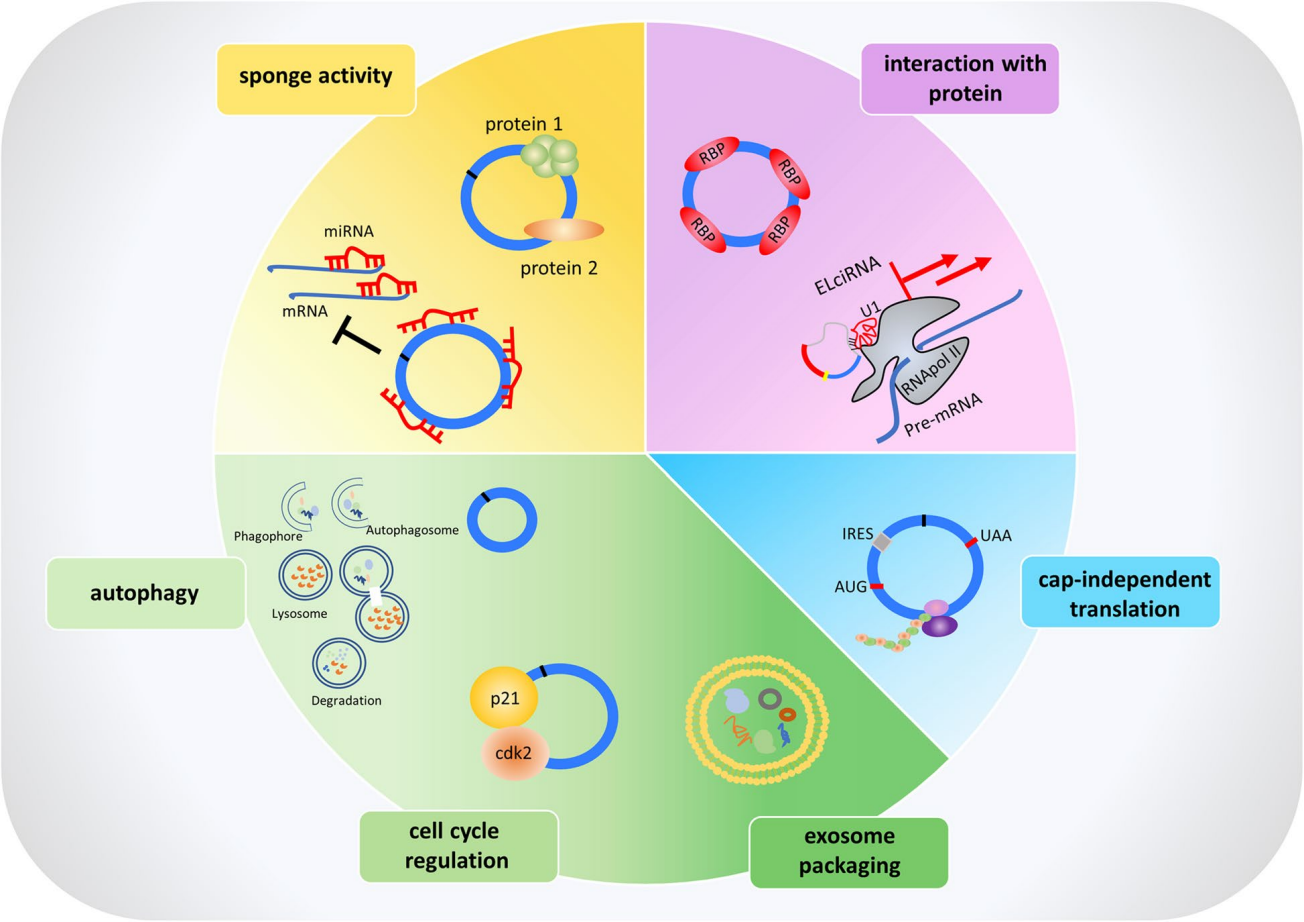
CircRNAs mechanisms of action. CircRNAs can act as miRNAs sponges inhibiting the silencing activity of miRNAs on their target genes and can also sponge proteins. They can interact with RNA binding proteins or with RNApol II and U1 splicing factor. They can undergo cap independent translation encoding small peptides. CircRNAs can be packaged into exosomes or can be involved in cell cycle regulation since they can act as scaffolding for proteins involved in cell cycle or can regulate autophagy process

Circular RNAs have been found to exert their functions through different mechanisms (Fig. 2). They can act as miRNA sponges (Fig. 2). Indeed, they present binding sites for particular miRNAs and modulate gene expression by inhibiting the silencing activity of miRNAs on their mRNAs target [16]. For example, the circular RNA ciRS7 has about 70 binding sites for miR-7, repressing the function of the miRNA which is involved in cell proliferation and apoptosis acting as both oncogene and tumor-suppressor [17]. Besides this, circular RNAs can regulate the stability of mRNAs or can interact with RNA binding proteins (Fig. 2). Circ-foxo3 has been demonstrated to have a binding affinity for particular transcriptional factors related to stress and senescence such as Id-1 (Inhibitor of DNA binding protein 1), E2F2 (E2F Transcription factor 2), HIF-α (Hypoxia inducible factor) and FAK (Focal adhesion kinase), supporting the linear correlation between high levels of the circRNA molecule and senescence [18]. Furthermore, circRNAs can sponge proteins modulating their activity (Fig. 2). Circ-foxo3 has been demonstrated to interact with p21 (Cyclin-dependent kinase inhibitor 1) and CDK2 (Cyclin-dependent kinase 2) arresting cell cycle progression (Fig. 2) [19]. They have also been found to recruit proteins at a particular site acting as a scaffolding circRNA. Circ-Amotl1, is able to facilitate pyruvate dehydrogenase kinase 1 (PDK1) dependent phosphorylation of AKT serine/ threonine kinase 1 (AKT1) [8]. Of note, the exon-intron containing circular RNAs (EIciRNAs) are involved in regulating transcription forming a complex with RNA polymerase II by binding to the gene promoter. EIciRNAs have been shown to participate also in the splicing process since they can form complexes with U1 snRNP and polymerase II (Fig. 2) [20]. Moreover, recent studies of Legnini L. et al. and Pamudurti N. R. et al. showed that endogenous circular RNAs can be translated [21, 22]. Generally, only the linear mRNAs are translated because they present the 5’ end cap structure, but it has been demonstrated that some circular RNAs can be translated in a 5’ end cap independent manner because of the presence of internal ribosomal entry sites (IRES) (Fig. 2) [23]. This is the case of circMbl3, which was translated in flies, [22] and circ-ZNF609, whose protein product has been proposed to be involved in myogenesis [21].

Interestingly, circRNAs can also be packaged into exosomes, the extracellular vesicles that mediate the transport of circRNAs even to nearby cells or body fluids, this can be exploited as biomarkers for cancer diagnosis (Fig. 2) [24].

Emerging evidence pointed out the importance of circular RNA in various diseases, such as in cancer [25] where it was reported that circRNAs can act also as autophagy regulators [26] (Fig. 2). Notably, recent studies proposed that circular RNAs analyses can contribute towards better understanding cancer progression and moreover can be used as novel biomarkers [27].

**Fig. 3:**
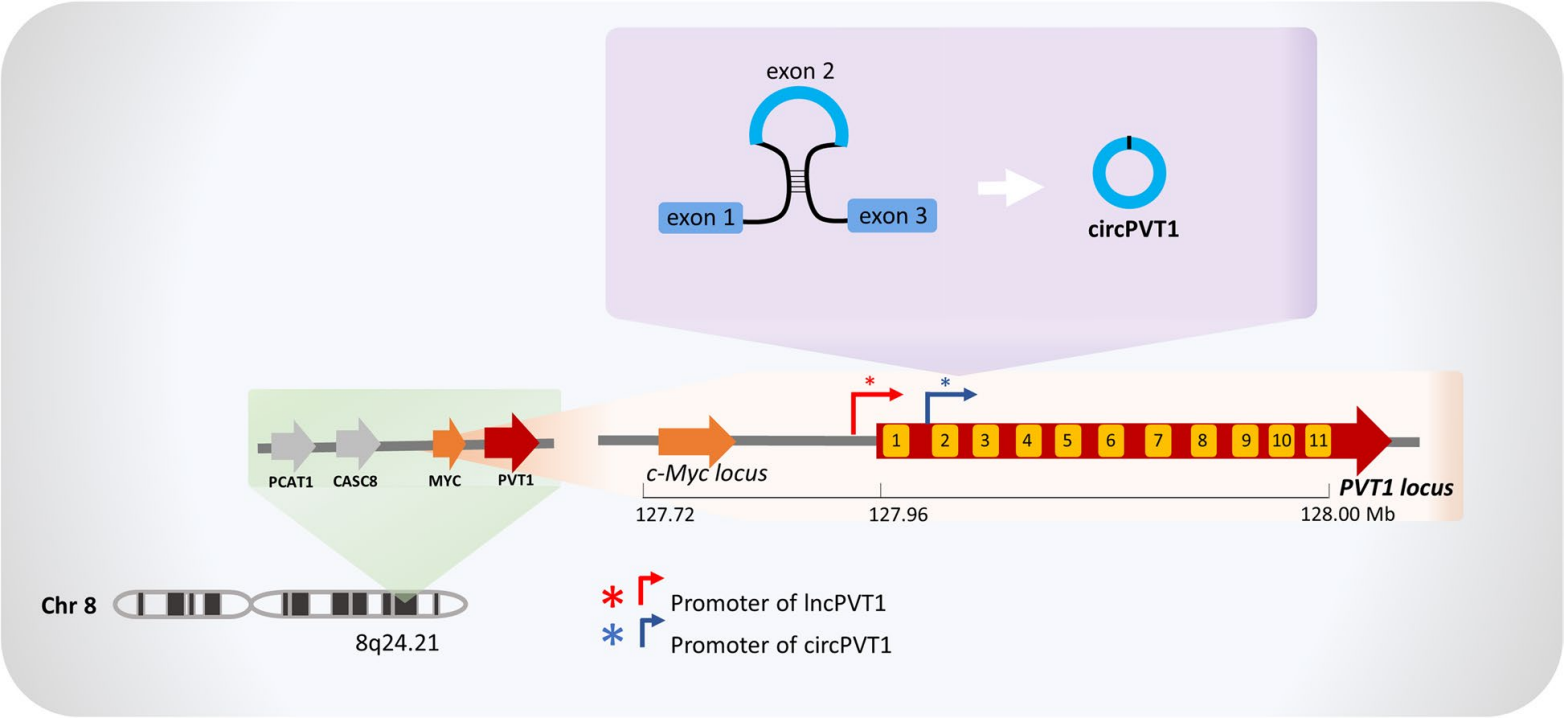
PVT1 locus. PVT1 is located in a cancer susceptibility locus on chromosome 8, near c-MYC locus, and encodes for both long non-coding PVT1 and circPVT1 molecules, the latter derives from back-splicing of exon 2. Long non-coding PVT1 and circPVT1 molecules appear to be transcribed from two independent promoters

Among the different circular RNAs involved in cancer, circularPVT1 has been shown to have important oncogenic features in several types of cancer [28] and a potential role as prognostic biomarker [29]. Notably, its transcription site is located in a cancer susceptibility locus. The circRNA molecule originates from the PVT1 locus that encodes a lncRNA called lncPVT1, a well-known long non-coding RNA involved in cancer development and progression (Fig. 3). Moreover, it is located at 53 Kb from c-MYC locus, which is often upregulated in cancer and even if an independent transcription of circPVT1 and lncPVT1 by two different promoters was reported [28], different sets of evidence have highlighted a potential interaction between them since they share the same targets [30]. In the following sections, the oncogenic role of circPVT1 focusing on its "paradigmatic" network interaction is described.

## The circPVT1/lncPVT1/c-MYC oncogenic network

Circular PVT1 was first identified as a senescence suppressor [31] and then as a proliferative factor in gastric cancer [32]. It derives from the circularization of exon 2 of the Plasmacytoma variant translocation 1 PVT1 gene in which the presence of Alu repeats in the flanking introns seems to favor the circularization process [33].

PVT1 is located on the 8q24.21 region recognized as a cancer susceptibility locus (Fig. 3) [34]. It can present aberrant translocations, viral integrations and amplifications [35]. Moreover, the two fragile sites FRA8C and FRA8D are located in this locus promoting genome instability and activation of oncogenes. Under particular conditions that can affect DNA replication such as hypoxia, these fragile sites induce chromosome breakage [36].

PVT1 encodes for a lncRNA, called lncPVT1 that has been shown to be associated with several tumors as well as the development of chemoresistance [34]. Interestingly, it is located only 53 Kb downstream c-MYC, the oncogene that regulates the expression levels of about 15% of all genes [37].

PVT1 and c-MYC belong to the same topologically associated domain (TAD) and the PVT1 promoter seems to play an independent role compared to lncPVT1. Notably, Cho S.W and colleagues showed that the promoter of lncPVT1 acts as a tumor suppressor DNA boundary element thus finely tuning the expression of c-MYC. Since they are located in the same TAD, promoter competition has emerged among them. In particular, a competitive dynamic for binding between enhancers and the E-boxes present in the c-MYC and PVT1 promoters takes place [38]. Usually, the enhancers preferentially bind the E-boxes on the PVT1 promoter hindering c-MYC transcription. In human cancers mutations present in the PVT1 promoter alter the tridimensional structure of the TAD and allow the enhancers to promote c-MYC transcription favoring tumor progression [39].

On the other hand, a positive feedback loop among lncPVT1 and c-MYC has been reported. LncPVT1 acts at a post-translational level on the stability of c-myc preventing its degradation through the proteasome pathway [40]. Moreover, this lncPVT1-c-myc duet is supported also by c-myc that shuttles into the nucleus and binds PVT1 promoter favoring its transcription [41].

Interestingly, there are several sets of evidence showing that two different promoters drive independently lncPVT1 and circPVT1 expression. Indeed, Verduci and colleagues analyzed the intronic region upstream the exon 2 from which circPVT1 originates and found a TATA box binding site, indicating a putative promoter region. Moreover, the TEAD/YAP/mut-p53 complex that they discovered to be involved in circPVT1 mechanism of action was found by chromatin immunoprecipitation on the circPVT1 promoter [42].

After first identifying circPVT1 in gastric cancer, further analyses have unveiled circPVT1’s oncogenic role, as well as lncPVT1/c-MYC’s role in other types of cancer such as osteosarcoma [43], head and neck squamous cell carcinoma [42], non-small cell lung carcinoma [44], acute lymphoblastic leukemia [45], esophageal carcinoma [46], colorectal carcinoma [47] and hepatocellular carcinoma [48]. Even if recent evidences show an involvement of circPVT1 also in oral squamous cell carcinoma [49], epithelial ovarian cancer [15], breast cancer [50] and prostate cancer [51]. More detailed information is reported in Table 1.

**Table 1:** Update list of evidences about the role of circPVT1 in tumor malignancies

Disease (cancer)	Target miRNA	Downstream effector	References
**Gastric cancer**	miR-125b	E2F2	[52]
	miR-124-3p	ZEB1	[53]
	miR-30a-5p	YAP1	[54]
	miR-152-3p	HDGF	[55]
**Osteosarcoma**	miR-205-5p	c-FLIP	[56]
	miR-526b	FOXC2	[57]
	miR-137	TRIAP1	[58]
	miR-423-5p	Wnt5a/Ror2 and c-FLIP	[56]
**Head and neck squamous cell carcinoma**	miR-497-5p	Aurka, mki67, bub1	[42]
	miR-106a-5p	Hexokinase II	[49]
**Non-small cell lung cancer**	miR-125b	E2F2	[59]
	miR-497	Bcl-2	[60]
	miR-30d/e	CCNF	[61]
	Let-7	NRAS	[62]
	miR-429	FOXK1	[63]
	miR-145-5p	ABCC1	[64]
**Acute lymphoblastic leukemia**	Let-7	c-MYC	[65]
	miR-125	Bcl-2	
	miR-30e	DDL4	[66]
**Colorectal carcinoma**	miR-145	PAK4	[47]
**Hepatocellular carcinoma**	miR-203	HOXD3	[67]
	miR-3666	SIRT7	[68]
	miR-377	TRIM23	[69]
**Esophageal carcinoma**	miR-4663	Pax-4, Pax-6	[46]
**Breast cancer**	miR-204-5p	EMT	[50]
	miR-29a-3p	AGR2	[70]
**Renal cell carcinoma**	miR-145-5p	TBX15	[71]
**Thyroid cancer**	miR-455-5p	CXCL12/CXCR4	[72]
**Gallbladder cancer**	miR-339-3p	MCL-1	[73]

In the following section, we describe the circPVT1/lncPVT1/c-MYC mechanisms through which they exert their oncogenic activities in different tumors, either in combination or alone, in more detail.

### Gastric cancer

Gastric cancer (GC) represents the fourth most common cancer worldwide and is etiologically associated with Helicobacter pylori infection as well as lifestyle factors, diet and genetics which also contribute to the development of this cancer [74]. Circular PVT1 was found upregulated in gastric cancer and correlated with tumor progression and poor prognosis [52]. Chen J. and colleagues reported that the circular RNA molecule was mainly localized within the cytoplasm compared to the nuclear localization of its linear counterpart lncPVT1, where it exerts its oncogenic activity by acting as a sponge for the miR-125 family, thus allowing the overexpression of the pro-proliferative factor, E2F2 [52, 59].

CircPVT1 overexpression was reported to be involved in paclitaxel resistance since it was able to regulate the expression of Zinc finger E-box binding homeobox 1 (ZEB1), an inhibitor of E-cadherin proteins, by sponging miR-124-3p, which is usually downregulated in paclitaxel resistant GC cells [53, 75]. Moreover, Yao W. and colleagues showed that circPVT1 was also associated with cisplatin resistance in GC [54]. Indeed, circPVT1 overexpression was demonstrated to directly target miR-30a-5p, leading to the overexpression of Yes-associated protein 1 (YAP1) which is related not only to the proliferation and inhibition of apoptosis but also to drug resistance [54]. A recent study by Sui H. and colleagues showed that Propofol, which is used in cancer surgery for anesthesia, suppressed proliferation and invasion of GC impinging on the circPVT1/miR-195-5p/ETS1 axis [76]. Notably, the downregulation of circPVT1 allowed miR-195-5p to inhibit the ETS Proto-Oncogene 1 (ETS1), in which aberrant expression correlates with deregulated cell proliferation, invasion and metastasis [77].

Recent studies by Wang X. and colleagues also investigated the role of circPVT1 in cisplatin resistance gastric cancer cells demonstrating that the circRNA molecule regulates malignancy of cisplatin chemoresistance gastric cancer cells via miR-152-3p [55]. Indeed, this miRNA results to be downregulated in chemo-resistant cells while its target gene the Hepatoma-derived growth factor which is upregulated, is implicated in resistance and proliferation [55]. Previously, it was reported that also lncPVT1 acts as a sponge for miR-152-3p regulating its function in gastric cancer [78]. These sets of evidence cannot exclude the possible synergistic effect even if in the study of Chen J. and colleagues circPVT1 and PVT1 were poorly correlated [52].

### Head and neck cancer

The oncogenic role of circPVT1 was identified also in head and neck squamous cell carcinoma (HNSCC), a heterogeneous type of tumor that develops in the epithelial cells of the mucosal borders of the upper airways and food tract [79]. Verduci L. and colleagues demonstrated that circPVT1 expression level, but not lncPVT1, was upregulated in HNSCC and significantly correlated with the presence of mutated p53 [42], which is known to be associated with a poor overall survival and locoregional recurrence in HNSCC [80]. The authors found that circPVT1 overexpression was due to the activity of mut-p53/YAP/TEAD complex. Chromatin immunoprecipitation (ChIP) assays revealed that mut-p53 and TEAD (TEA domain transcription factor 1) were recruited at circPVT1 promoter region where a TEAD consensus sequence, which allows the binding of YAP, was present promoting transcription of circPVT1. YAP was also involved in the post-transcriptional regulation of circPVT1. Interestingly, circPVT1 bound YAP to maintain its own expression levels [42]. The same authors pointed out that circPVT1 acts as a sponge for miR-497-5p thus inhibiting the translation of miR-497-5p targets such as Aurka (Aurora kinase A), mki67 (Marker of proliferation ki67) and bub1 (Budding uninhibited by benzimidazoles 1) which are involved in cell proliferation.

A recent study by Zhu X. and colleagues revealed that circPVT1 was involved in the oncogenesis of oral squamous cell carcinoma (OSCC). In that context, circPVT1 was found to sponge the tumor-suppressor miR-106a-5p leading to an overexpression of the miRNA target Hexokinase II (HK2), a glycolytic enzyme upregulated in cancer [49].

Interestingly, in nasopharyngeal carcinoma (NPC) Cui M. and colleagues found that also the lncPVT1 is upregulated in cancer cells promoting proliferation, stem-cell phenotype by negatively regulating miR-1207 expression [81]. The high expression of lncPVT1 also correlates with worse progression-free and overall survivals suggesting that it could be considered a potential oncogene [82]. Yu C. and colleagues demonstrated also that lncPVT1in squamous cell carcinoma is able to promote proliferation and invasion by activating Wnt/β- catenin signaling pathway suggesting that the lncRNA molecule plays the role of a therapeutic target [83].

### Osteosarcoma

Osteosarcoma (OS) represents the most frequent solid tumor of the bones and it is characterized by the presence of mesenchymal cells that produce osteoid and/or immature bone [84, 85].

Recent studies by Kun-Peng and colleagues revealed that circPVT1 could be a potential biomarker for osteosarcoma [43]. Notably, they showed that circPVT1 was highly upregulated in OS, where it promotes chemoresistance to doxorubicin and cisplatin through decreasing the expression of ATP-binding cassette subfamily B member 1 (ABCB1), one of the most important proteins involved in multidrug resistance [43, 86]. Moreover, they found that the assessment of circPVT1 serum levels was more useful for OS diagnosis compared to alkaline phosphates (ALP) and lactate dehydrogenase (LDH), the most commonly used biomarkers in osteosarcoma to date.

Interestingly, Liu Y. and colleagues reported that silencing circPVT1 in osteosarcoma cell lines decreased the expression of N-cadherin (Neural cadherin), Vimentin and Snail while it increased the expression of E-cadherin (Epithelial cadherin) suggesting circPVT1’s role in epithelial-mesenchymal transition (EMT). They also showed that circPVT1 exerts sponge activity on miR-205-5p that targets c-FLIP (Cellular FLICE-inhibitory protein) which is an anti-apoptotic regulator [56, 87]. Recent studies by Yan M. and colleagues revealed that circPVT1 is associated with OS cell metastases through the miR-526b/FOXC2 axis. They found that circPVT1 knockdown suppressed migration and invasion of OS cells. In order to further investigate how circPVT1 promotes metastases, the same group showed that circPVT1 was able to upregulate FOXC2 (Forkhead box C2) expression, a transcriptional factor belonging to the forkhead/winged-helix family which has been demonstrated to be involved in the epithelial-mesenchymal transition by modulating cadherin protein expression and other molecules involved in the EMT process [57]. They also demonstrated that circPVT1 is able to act as a sponge for miR-525b contributing indirectly to FOXC2 overexpression, a well-known tumor angiogenesis promoter [57]. Besides this, recent studies by Wang J. and Liu YP’s group showed that circPVT1 contributes to inducing glycolysis and metastasis by sponging miR-423-5p and miR-205-5p thus activating Wnt5a/Ror2 and c-Flip signaling pathways, respectively [56, 88]. Study of Li D. and colleagues demonstrated that circPVT1 sustains doxorubicin resistance in osteosarcoma by acting as a sponge for miRNA-137 leading to the upregulation of its target gene TP53-regulated inhibitor of apoptosis 1 (TRIAP1) suggesting that circPVT1 could be used as a promising therapeutic target of osteosarcoma treatment [58].

While all these bodies of evidence did not check lncPVT1 levels in the same experimental settings, there are several other studies that demonstrated the role of the long non-coding in osteosarcoma. Notably, Zhou Q. and colleagues showed that also lncPVT1 results upregulated in osteosarcoma cancer cells involved in cancer development by regulating miR-195 [89]. Furthermore, Song J. and colleagues revealed that high lncPVT1 expression correlates with poor prognosis and induces the expression of hexokinase II via sponging the miR-497 which normally exerts a suppressive effect on glycolysis [30]. Yan M. and colleagues revealed that lncPVT1 is also involved in metastases by acting as a molecular sponge for miR-484 which results to be downregulated in osteosarcoma cancer cells [90], while an additional study showed that lncPVT1 is involved in enhancing chemoresistance to gemcitabine by promoting the activation of c-MET/PI3K/AKT pathway [91].

Considering all these lines of evidences, it seems evident how both circPVT1 and lncPVT1 overexpression promote osteosarcoma development, progression and chemoresistance. Accordingly, they might represent a potential interesting target in molecular treatment of osteosarcoma in order to suppress cancer cell proliferation and metastases**.**

### Non-small cell lung cancer

Lung cancer is the leading cause of cancer death in developed countries with 16.6% of 5-year survival rate and where non-small cell lung cancer (NSCC) is the predominant subtype of lung cancer [92, 93].

Li X. and colleagues revealed that circPVT1 results upregulated in NSCLC, where it promotes cell proliferation and invasion [33]. In particular, they found that c-Fos (Fos proto-oncogene) promotes circPVT1 expression, transcriptionally. Chromatin immunoprecipitation (ChIP) assay revealed an enrichment of c-Fos at the level of circPVT1 promoter region. It is important to note that the knockdown of circPVT1 affected cell proliferation and invasion in these experimental settings, confirming the hypothesis that circPVT1 acts as an oncogene in NSCLC [33].

Two lines of evidence revealed how circPVT1 oncogenic activities are also related to the E2F2 signaling pathway, which is usually involved in cell cycle regulation, differentiation and tumorigenesis [94]. CircPVT1 is able to promote E2F2 by acting as a competitive endogenous RNA for miR-125b that directly targets E2F2 [59]. Notably, RNA immunoprecipitation (RIP) assay after circPVT1 silencing showed an enrichment of miR-125b, thus indicating a possible direct binding between circPVT1 and miR-125b. Moreover, circPVT1 overexpression promoted a lowering of miR-125b levels, thus restoring pro-tumorigenic E2F2 activities [33].

Qin S. and colleagues demonstrated that circPVT1 exerts a sponge activity on miR-497 which targets the anti-apoptotic protein Bcl-2 (B-cell lymphoma 2) [60]. Indeed, they showed that circPVT1 plays an oncogenic role in NSCLC by indirectly controlling the anti-apoptotic protein Bcl-2 expression through miR-497 binding.

Interestingly, circPVT1 seems to also be involved in chemotherapy resistance. In lung adenocarcinoma (LAD) that represents the main subtype of NSCLC, Zheng F. and colleagues showed that circPVT1 upregulation is associated with cisplatin and pemetrexed resistance [64]. Upon circPVT1 silencing LAD cell lines become more sensitive to cisplatin and pemetrexed [64]. Notably, circPVT1 exerts its oncogenic activity by targeting miR-145-5p leading to the upregulation of the miR-145-5p target responsible for multi-drug resistance [64]. Interestingly, NSCLC patients who received cisplatin combined with gemcitabine treatment showed a decrease in circPVT1 expression while those patients belonged to the chemotherapy-resistant group expressed a higher level of the circRNA molecule, thus suggesting the idea that by detecting the expression level of circPVT1 in serum it is possible to evaluate the chemotherapy outcome [95]. Recent study of Huang M. and colleagues showed that circPVT1 is upregulated in cell upon irradiation treatment highlighting that silencing of circPVT1 enhances NSCLC cell sensitivity response by acting as a sponge for miRNA-1208 [96]. Recent study of Shi J. and colleagues, demonstrated that circPVT1 promotes the tumorigenic phenotype in lung squamous cell carcinoma by sequestering miR-30d and miR-30e thus upregulating their target CCNF (Chromatin Condensation and Nuclear Fragmentation), the substrate recognition subunit of SCF ubiquitin ligase complexes which has a pivotal role in cancer since it is able to sustain cell proliferation [61]. Another study of Danac J. M. C. and colleagues showed that circPVT1 upregulates NRAS (Neuroblastoma RAS viral oncogene homolog) by acting as a miRNA sponge on let-7 thus sustaining the oncogenic phenotype [62]. Cao L. and colleagues recently demonstrated that in lung adenocarcinoma, circPVT1 results upregulated and associated with cisplatin resistance [63]. They found that circPVT1 exerts an oncogenic activity by sequestering miR-429 leading to the upregulation of the miRNA target FOXK1 (forkhead box k1), important regulator of cell proliferation. Indeed, knockdown of circPVT1 inhibits proliferation, migration and invasion of adenocarcinoma cells and also increase cisplatin sensitivity [63].

Non-small cell lung cancer is not only characterized by circPVT1 upregulation, several studies showed that also lncPVT1 is upregulated in cancer cells and contributes to cell proliferation, apoptosis, migration and invasion through different mechanisms among which the most frequent acts as a molecular sponge for miRNAs. Qiu C. and colleagues reported that lncPVT1 is able to enhance cancer progression by targeting miR-526b leading to the upregulation of its target EZH2 (Enhancer of zeste homolog 2) which stimulates proliferation, where it is also a prognostic marker [97]. Mao Z. and colleagues found that lncPVT1 is able to promote angiogenesis by regulating VEGF (Vascular endothelial growth factor) expression levels by sponging miR-29c [98].

### Colorectal cancer

Colorectal cancer (CRC) represents one of the most frequent causes of cancer-related death worldwide and is the third most common cancer type found in the US affecting both males and females. Among the factors that are associated with colorectal cancer development can be mentioned age, genetic and environmental factors but also the presence of a family history of colon cancer [99]. An important role in the incidence of colorectal cancer is played by alcohol consumption [100] and obesity [101], both conditions increase the risk of developing colorectal cancer. Colorectal cancer is usually diagnosed at a late stage and distant liver and/or lung metastases are often present [102]. Wang Z. and colleagues showed that circPVT1 could be used as a potential biomarker and as a molecular target for colorectal cancer treatment [47]. Indeed, they demonstrated that circPVT1 results upregulated in 92.19% of the 64 CRC tumor tissues that were considered in the study [47]. The same authors also demonstrated how circPVT1 knockdown suppresses migration as an invasive feature. In particular, they found that circPVT1, mainly localized in the cytoplasm, acted as a competing endogenous RNA for miR-145. The latter is a tumor suppressor microRNA that is usually downregulated in CRC and is associated with tumorigenesis and migration [47]. Indeed, among its target genes there is the p21-activated kinases 4 (PAK4) which plays a pivotal role in cell cytoskeletal reorganization that is required for cell migration [103]. Interestingly, other studies revealed that also the lncPVT1 is upregulated and is involved in the progression of CRC via apoptosis inhibition [104]. A study of Liu F. and colleagues showed that inhibition of lncPVT1 expression was able to suppress cancer progression acting on the miR-106b-5p/FJX1 axis. In colorectal cancer cells where lncPVT1 is upregulated, it exerts a sponge activity on miR-106b-5p and consequently leading to the aberrant expression of the miRNA target FJX1 (Four jointed box 1) that is involved in cancer progression [105].

### Hepatocellular carcinoma

Hepatocellular carcinoma (HCC) represents more than half of the total liver cancers and is usually related to cirrhosis that results after hepatitis B (HBV) and C virus (HCV) infection or can be related also to an alcoholic injury or to genetic disorders [106, 107]. It is rarely diagnosed before the age of 40, while it is very frequently found at 70 years of age (Hashem B. et al., 2002). Recently, Zhu Y. and colleagues showed that circPVT1 could be a potential drug target for hepatocellular carcinoma, since the circRNA molecule results to be upregulated in the tumour tissues and correlates with poor prognosis [108]. In order to better evaluate the potential oncogenic effect of circPVT1 in HCC, the same group performed circPVT1 silencing and overexpression analyses. Interestingly, circPVT1 has been found to bind miR-203, thus restoring the expression of HOXD3 (Homeobox D3) [67]. HOXD3 is the third paralogue of HOXD gene family and it has been demonstrated to be involved in the modulation of N-cadherin, integrins and E-cadherin expression, as well as vascular epithelial growth factor receptor (VEGFR) expression [109]. Wang L. and colleagues showed that HOXD3 results upregulated in HCC, where it promotes angiogenesis by directly regulating VEGFR expression [67]. The development of targeted molecular therapies that directly impinge on circPVT1 expression could restore the oncosuppressor activities of miR-203 in HCC.

A very recent study by Li Y. and colleagues revealed that circPVT1 exerts a sponge activity also on the tumour suppressor miR-3666 which is downregulated in HCC. Indeed, circPVT1 via miR-3666 binding regulates the downstream miRNA target Sirtuin 7 (SIRT7), a potential oncogenic protein which results upregulated in HCC [68, 110]. Interestingly, circPVT1 overexpression promotes SIRT7 upregulation altering the p53 cell death pathway since SIRT7 is able to inhibit p53-mediated apoptosis [111]. Notably, circPVT1 seems to exert its oncogenic activity in HCC by acting on the miR-3666/SIRT7 axis supporting in this way the HCC cell growth modulating p53 activity. Another recent study conducted by Bu N. and colleagues showed that circPVT1 also plays an oncogenic role by acting as a competitive endogenous RNA that sponges miR-377 leading to the upregulation of the target gene TRIM23 (Transcripts encoding tripartite motif containing 23), a ubiquitin ligase that was established to be a regulator of carcinogenesis inducing in particular migration and invasion [69, 112]. Alternatively, other studies were conducted regarding the expression of the lncPVT1 in hepatocellular carcinoma and Guo J. and colleagues showed that it results upregulated and mediates the recruitment of EZH2 in order to stabilize mdm2 (Mouse double minute 2 homolog ) protein that in turn inhibits p53 expression leading to apoptosis inhibition [113].

### Esophageal cancer

Esophageal cancer (EC) represents one of the most aggressive squamous cell carcinomas. The majority of EC patients exhibit metastasis at diagnosis, which dramatically impacts on treatment outcome [114, 115].

Zhong R. and colleagues showed that circPVT1 results overexpressed in esophageal carcinomas. Interestingly, they found that circPVT1 expression correlated with increased cell proliferation while, depletion of circPVT1 reduced survival of EC cells [46]. Moreover, the overexpression of circPVT1 was associated with an increased invasive ability of EC cells suggesting a strong association of circPVT1 expression and cancer phenotype [46]. In particular, they discovered that circPVT1 modulates the protein expression levels of paired box genes Pax-4 and Pax-6 through miR-4663 binding. The latter promotes tumorigenesis and modulates the expression of proliferation-activated receptors (PPARs) which inhibit tumor growth [46].

Xu Y. and colleagues investigated the role of lncPVT1 in esophageal adenocarcinoma and reported that the high expression of lncPVT1 was associated with poor differentiation, lymph node metastases and short overall survival [116]. Moreover, Li P. and colleagues revealed that lncPVT1 supports the oncogenic phenotype by acting as a molecular sponge for miR-203 leading to LASP1 (LIM And SH3 Protein 1) upregulation involved in migration and invasion [117]. Indeed, downregulation of the lncPVT1 molecule suppressed cell viability, migration and invasion inducing apoptosis through the disabling of the sponge interaction involving lncPVT1 with miR-145 [118].

### Breast cancer

Breast cancer (BC) represents the most common type of cancer that occurs in women. Although, innovative methodologies and treatment have significantly increased the overall survival, the identification of new molecular targets, as well as new diagnostic biomarkers could be of critical importance. Bian Q. and colleagues reported that circPVT1 acts as oncogenic factor in breast cancer by promoting migration and invasion of breast cancer cells in vitro. Its levels were also found upregulated in tumor tissues and correlates with poor prognosis. Intriguingly, circPVT1 was able to promote epithelial to mesenchymal transition serving as a competing endogenous RNA for miR-204-5p [50]. Moreover, another recent study of Wang J. and colleagues demonstrated that circPVT1 which resulted upregulated in breast cancer, exerts a sponge activity on miR-29a-3p leading to the upregulation of AGR2 (Anterior gradient protein 2) that in turns enhance HIF-1α expression accelerating cell proliferation, migration and invasion abilities of breast cancer cells [70].

Further investigation regarding circPVT1’s role in breast cancer is needed in order to best dissect the role of circPVT1 in breast cancer oncogenesis as well as the role of the lncPVT1 needs to be additionally investigated. Indeed, several studies showed that lncPVT1 results upregulated in breast cancer [67, 119, 120]. Interestingly, Tang J. and colleagues investigated the role of lncPVT1 in triple negative breast cancer demonstrating that lncPVT1 upregulation correlates with tumorigenicity through the activation of the beta-catenin pathway, having a critical role in cell proliferation and metastases [120]. Another study conducted by Wang L. and colleagues showed that lncPVT1 exerts its oncogenic activity also through the modulation of p21 expression level, while Liu X. and colleagues reported the miR-1204, that is located in the PVT1 locus, results to be upregulated and correlated with cell proliferation, migration, invasion and epithelial mesenchymal transition [119].

### Prostate cancer

Prostate cancer represents one of the most common cancer types that develop in the male reproductive system. Even if existing therapeutic solutions such as radical prostatectomy or radiation are adopted, the majority of the patients develop disease recurrence [121]. Interestingly, Yang J. and colleagues reported that long non-coding PVT1 is involved in prostate cancer, since its expression is upregulated in the cancer tissues, and correlates also with poorer overall survival and disease-free survival [121]. Similarly, Pal G. and colleagues showed that the long non-coding from exon 9 of PVT1 favors tumorigenesis by promoting cell proliferation and migration through the regulation of proliferating cell nuclear antigen (PCNA) expression [122]. Umemori M. and colleagues very recently reported a strong functional interplay between the oncogene c-MYC and circPVT1. Indeed c-MYC expression was strongly reduced only after depletion of circPVT1 and not of lncPVT1 expression, highlighting a possible circPVT1 involvement in the stabilization of c-myc protein [51]. In conclusion, investigating the PVT1 locus could be very promising for a deeper understanding of prostate cancer development with potential therapeutic implications.

### Acute lymphoblastic leukemia

CircPVT1 has been also found to promote acute lymphoblastic leukemia (ALL), a heterogeneous lymphoid neoplasm that derives from T- and B-lymphoid progenitors and represents one of the most diffused tumors in pediatric age [123]. Indeed, circPVT1, but not lncPVT1, was found upregulated in ALL patients and its expression associates with higher cell proliferation and tumor progression [45]. Interestingly, Ghetti M. and colleagues demonstrated that acute lymphoblastic leukemia is characterized by the presence not only of PVT1 amplification but also by PVT1 fusion genes (chimeras). In particular, they reported that c-MYC results to be upregulated and correlated with PVT1, while knockdown of c-MYC led to a downregulation of PVT1 [65]. Moreover, the same downregulation of the circPVT1 was able to decrease cell proliferation and to promote apoptosis through the downregulation of c-myc and bcl2 protein expression. Indeed, since circPVT1 is localized in the cytosol, it exerts its sponge activity on miRNAs let-7 and mir-125 that target c-MYC and BCL2, respectively [65]. This study reveals the complexity of the interaction network between circPVT1/lncPVT1 and c-MYC and why they should be investigated concomitantly to better understand their pro-tumorigenic activities. Recent study of Jia Y. and colleague demonstrate that the upregulation of circPVT1 activates the NOTCH signaling pathway through the miR-30e/DLL4 axis [66]. Indeed, circPVT1 acts as a miRNA sponge for miR-30e leading to the upregulation of its target gene DDL4 (Delta Like Canonical Notch Ligand 4) which is a ligand for NOTCH signaling that is involved in sustaining tumorigenesis [66]. Moreover, another recent study conducted by Jia Y. and colleagues reported that by sponging miR-125b, circPVT1 is able to enhance the NF-kappaB signaling pathway thus promoting cell proliferation and invasion [124].

### Other tumors

Recent studies have revealed circPVT1 implications also in renal cell carcinoma and thyroid cancer. Zheng Z. and colleagues demonstrated the oncogenic role of circPVT1 in renal cell carcinoma (RCC). They found that circPVT1 expression was significantly increased in patients’ serum. Moreover, they found that circPVT1 explicates its tumorigenic activity by directly interacting with miR-145-5p leading to the upregulation of one of its targets TBX15 (T-Box Transcription Factor 15), associated with the progression of RCC [71]. Intriguingly, other recent study by Zheng X. and colleagues revealed that circPVT1 is associated with medullary thyroid cancer, sustaining the metastatic process by acting on miR-455-5p/CXCL12/CXCR4 signaling axis [71]. CircPVT1 results to be involved also in gall bladder cancer (GBC) where Wang S. and colleagues demonstrated that it is upregulated and promotes proliferation, migration and invasion as well as inhibits cell apoptosis acting as a miRNA sponge for miR-339-3p thus upregulation its target MCL-1 (myeloid cell leukemia-1) which is an anti-apoptotic protein involved in cancer progression [73]. Moreover, a recent study by Wang YY and colleagues also revealed that circPVT1 is associated with adenomyosis, supporting endometrial cell proliferation and invasion by sequestering miR-145 [125]. Intriguingly, it has been reported also an oncogenic role of circPVT1 in acute myeloid leukemia [126].

## Conclusions

**Fig. 4:**
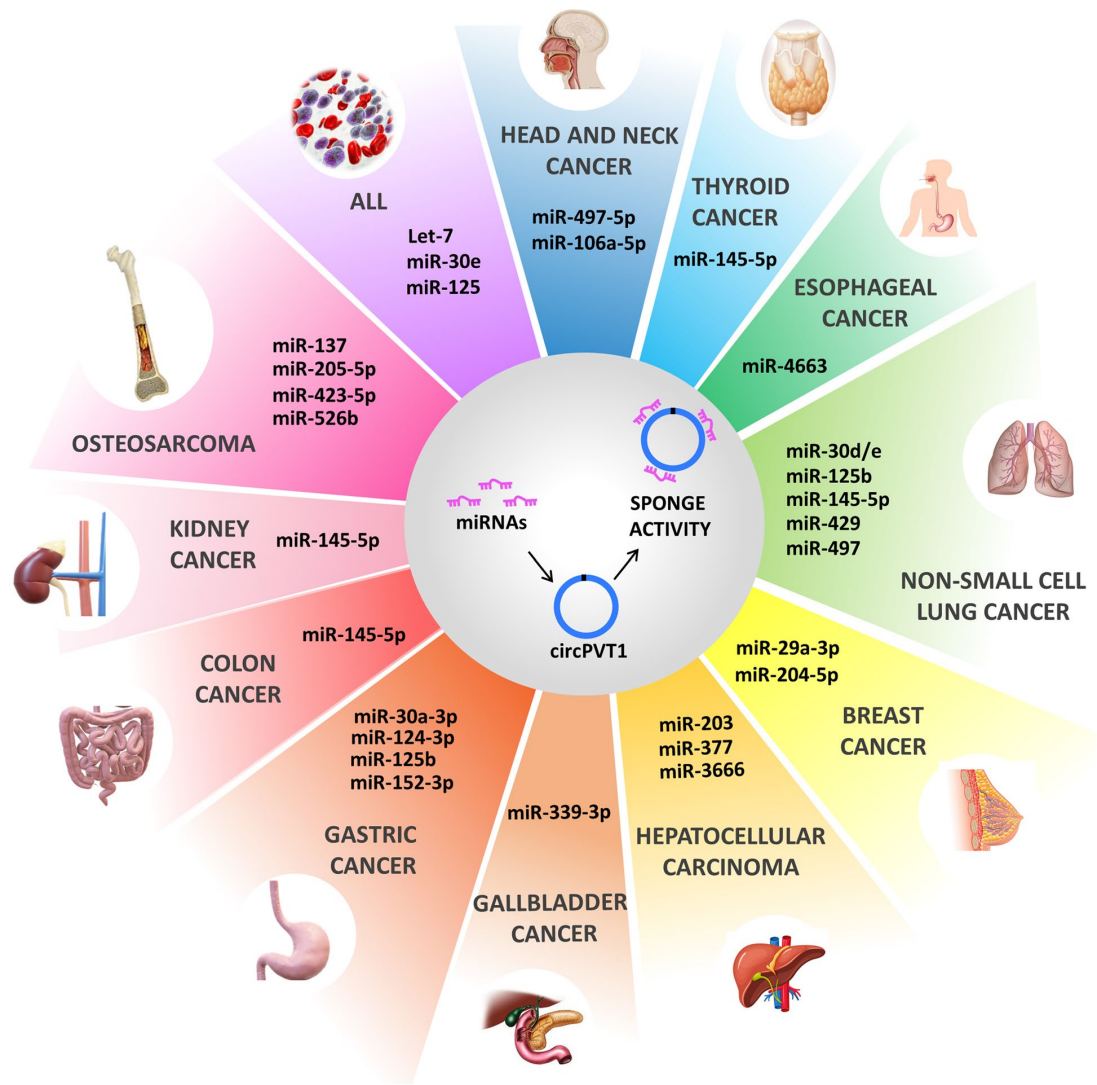
The oncogenic activity of circPVT1. CircPVT1 has been demonstrated to exert its action mainly by sponging oncosuppressor miRNAs, thus promoting tumorigenesis in different types of cancer

Here we report all known-published evidence regarding the role of circPVT1 in cancer (Fig. 4). Compared to its linear counterpart PVT1, circPVT1 involvement in other malignancies than those herein described still remains to be explored. Listed below are some of the unsolved issues that require additional experimental work to be deeply dissected:

- All the evidence reported so far point out how circPVT1 expression is associated with several tumorigenic features such as proliferation, invasion, angiogenesis and drug resistance. CircPVT1 is predominantly located in the cytosol, where it seems to act as a sponge for miRNAs. In particular, circPVT1 contains binding sites for several miRNAs, most of them are involved in tumor suppression by inhibiting translation of different oncogenes. Increasing evidences highlights the role of circPVT1 as a key player in oncogenesis. Accordingly, it could represent a potential target for the development of new precision drugs.
- Its circular structure makes circPVT1 resistant to RNAase enzymes action in body fluids, thus to represent an excellent potential diagnostic and prognostic biomarker to be included in liquid biopsy analyses.
- However, due to circPVT1 peculiar origin, it is necessary to study its pro- tumorigenic activities taking into account also its host gene, lncPVT1, and the presence of c-MYC. Only few of the reported studies have investigated this atypical network so far. Moreover, it is surprising that a circular RNA of only 202 nucleotides could promote such significant pro-tumorigenic effects only through the sponging of miRNAs.
- Until now, only one line of evidence provided by Tashiro and colleagues revealed that despite its non-coding origin, circPVT1 might encode for a protein of 104 amino acids. These findings need to be further validated but undoubtedly, they add a new level of complexity to the non-coding network instigated by the aberrant activity of circPVT1 [127]. Further studies are mandatory to better understand the role of circPVT1 in oncogenesis as well as its functional contribution to circPVT1/lncPVT1 and c-MYC axis in tumor progression.

## Data Availability

Not applicable.

## Abbreviations

ncRNAs: Non-coding RNAs
rRNAs: Ribosomal RNAs
tRNAs: Transfer RNAs
snRNAs: Small-nuclear RNAs
lncRNAs: Long non-coding RNAs
miRNAs: MicroRNAs
piRNAs: Piwi-associated RNAs
siRNAs: Small-interfering RNAs
circRNAs: Circular RNAs
circPVT1: Circular PVT1
lncPVT1: Long non-coding PVT1
Id-1: Inhibitor of DNA binding protein 1
E2F2: E2F Transcription factor 2
HIF- α: Hypoxia inducible factor
FAK: Focal adhesion kinase
PDK1: Pyruvate dehydrogenase kinase 1
AKT1: Serine/ threonine kinase 1
EIciRNAs: Exon-intron containing circular RNAs
IRES: Internal ribosomal entry sites
PVT1: Plasmacytoma variant translocation 1
TAD: Topologically associated domain
GC: Gastric cancer
ZEB1: Zinc finger E-box binding homeobox 1
YAP1: Yes-associated protein 1
TEAD: TEA domain transcription factor 1
ETS1: ETS Proto-Oncogene 1
HNSCC: Head and neck squamous cell carcinoma
Aurka: Aurora kinase A
mki76: Marker of proliferation ki67
Bub1: Budding uninhibited by benzimidazoles 1
NPC: Nasopharyngeal carcinoma
OS: Osteosarcoma
ABCB1: ATP-binding cassette subfamily B member 1
ALP: Alkaline phosphates
LDH: Lactate dehydrogenase
c-FLIP: Cellular FLICE-inhibitory protein
FOXC2: Forkhead box C2
TRIAP1: TP53-regulated inhibitor of apoptosis 1
NSCC: Non-small cell lung cancer
c-fos: Fos proto-oncogene
RIP: RNA immunoprecipitation
Bcl2: B-cell lymphoma 2
LAD: Lung adenocarcinoma
CCNF: Chromatin Condensation and Nuclear Fragmentation
FOXK1: Forkhead box k1
VEGF: Vascular endothelial growth factor
CRC: Colorectal cancer
PAK4: p21-activated kinases 4
FJX1: Four jointex box 1
HCC: Hepatocellular carcinoma
HOXD3: Homeobox D3
SIRT7: Sirtuin 7
TRIM23: Transcripts encoding tripartite motif containing 23
EZH2: Enhancer of zeste homolog 2
MDM2: Mouse double minute 2 homolog
EC: Esophageal cancer
LASP1: LIM and SH3 protein1
BC: Breast cancer
AGR2: Anterior gradient protein 2
ALL: Acute lymphoblastic leukemia
DDL4: Delta Like Canonical Notch Ligand 4
RCC: Renal cell carcinoma
TBX15: T-Box Transcription Factor
MCL-1: Myeloid cell leukemia-1
